# A Significant Question in Cancer Risk and Therapy: Are Antibiotics Positive or Negative Effectors? Current Answers and Possible Alternatives

**DOI:** 10.3390/antibiotics9090580

**Published:** 2020-09-06

**Authors:** Steffanie S. Amadei, Vicente Notario

**Affiliations:** Department of Radiation Medicine, Lombardi Comprehensive Cancer Center, Georgetown University Medical Center, Washington, DC 20057, USA; ss4406@georgetown.edu

**Keywords:** antibiotics, cancer risk, dysbiosis, gut microbiota, human tumor microbiome, symbiotic imbalances, therapeutic outcomes, tumor origin

## Abstract

Cancer is predominantly considered as an environmental disease caused by genetic or epigenetic alterations induced by exposure to extrinsic (e.g., carcinogens, pollutants, radiation) or intrinsic (e.g., metabolic, immune or genetic deficiencies). Over-exposure to antibiotics, which is favored by unregulated access as well as inappropriate prescriptions by physicians, is known to have led to serious health problems such as the rise of antibiotic resistance, in particular in poorly developed countries. In this review, the attention is focused on evaluating the effects of antibiotic exposure on cancer risk and on the outcome of cancer therapeutic protocols, either directly acting as extrinsic promoters, or indirectly, through interactions with the human gut microbiota. The preponderant evidence derived from information reported over the last 10 years confirms that antibiotic exposure tends to increase cancer risk and, unfortunately, that it reduces the efficacy of various forms of cancer therapy (e.g., chemo-, radio-, and immunotherapy alone or in combination). Alternatives to the current patterns of antibiotic use, such as introducing new antibiotics, bacteriophages or enzybiotics, and implementing dysbiosis-reducing microbiota modulatory strategies in oncology, are discussed. The information is in the end considered from the perspective of the most recent findings on the tumor-specific and intracellular location of the tumor microbiota, and of the most recent theories proposed to explain cancer etiology on the notion of regression of the eukaryotic cells and systems to stages characterized for a lack of coordination among their components of prokaryotic origin, which is promoted by injuries caused by environmental insults.

## 1. Bacterial Contributions to Eukaryotic Origins and Human Biology

In the continuous process through which living creatures kept progressively attaining levels of increasing structural and functional complexity, bacteria appeared much earlier than humans on the earth’s biosphere. Initially, bacterial populations interacted among themselves through mechanisms that contributed to increase their own diversity as well as their ability to colonize a wide range of environments. Then, as stated by the endosymbiosis theory [1,2], it was the stable symbiotic coalescing of different bacterial types that gave rise to a new type of more complex organisms, such as the earliest, unicellular eukaryotes. Since its first formal proposal over 50 years ago, although the endosymbiosis theory of eukaryogenesis has been challenged [3,4], reevaluated [5,6,7] and expanded [8,9,10], its validity has been largely supported [11,12]. Today, it is widely accepted that the symbiotic contributions of different structural and functional features by specialized prokaryotic organisms, particularly the mitochondria and chloroplast precursors, represent a fundamental transition that enabled eukaryotes to restructure their genomes and the acquisition of a tremendous bioenergetics potential for a much wider niche expansion, environmental adaptation, colonization and diversification, ultimately leading to the permanent establishment of organismal multicellularity.

In addition to setting up the foundation for the development of the enormous diversity and complexity attained by eukaryotic organisms, prokaryotic organisms, in general, and bacteria in particular, have provided another key beneficial role by coexisting with diverse host organisms, including humans, and establishing mutualistic interactions with them. In humans, large amounts of bacteria coexist in almost every organ and on all surfaces directly exposed to components of the external environment (e.g., skin, nasopharyngeal and oral cavities, lungs) or to byproducts derived from the digestion of dietary compounds (e.g., gastrointestinal system). The human endogenous commensal bacterial population most frequently studied and, therefore, the best characterized is what used to be called the “intestinal flora”, which more recently is indistinctively referred to as the gut “microbiota” or “microbiome”, although these terms are frequently confused and misused [13,14]. The term “microbiota” is correct when used to refer to the repertoire of strains of microorganisms in a given ecosystem [15]. The term “microbiome” is popularly believed to have been coined by Joshua Lederberg in the early 2000s [16]. In fact, the first microbiome notion was introduced in the 1800s by Sergey Winogradski, from a microbial ecology perspective, to refer to a microbial ecosystem (“microbe” plus “biome”) as a whole. Nevertheless, its current meaning departs from such a notion, and relates solely to the genomes of the microbial species inhabiting a particular ecosystem [17,18].

Over the last 10 years there has been an impressive renaissance in basic and clinical research related to the human microbiota and microbiome. The availability of improved culturing and sequencing methodologies [19,20,21], along with advances brought about by functional studies, have provided a wealth of knowledge on the role of the microbiota as a complex “organ” that performs essential roles in balancing health and disease states in humans [22,23,24,25]. The gut microbiota is known to establish a gut-organ network [26] that supports interactions with non-colonic microbiota [27] and with central homeostatic-regulatory controls, such as the immune system [28,29,30], the endocrine system [31], metabolism [32,33], the intrinsic circadian clock [34], brain function [35], and others. Through this network, the gut microbiota influences the onset, severity and outcome of diseases that cause high levels of morbidity and mortality among humans, including cardiovascular [36,37], liver [38], autoimmune [39] or infectious diseases [40], as well as cancer [41]. Although there are always nomenclature discontents [42], the terms eubiotic and dysbiotic, particularly the latter, have been used routinely to distinguish between the “good” (balanced in itself and with the host) and the “bad” (imbalanced) states of the gut microbiota [43,44]. While the distinction between the two states has been shown not to be a black-and-white case [45,46], it has been generally accepted that the beneficial and disease-promoting roles of the microbiota are associated with its eubiotic and dysbiotic stages, respectively [47,48].

Some microbes coexisted and coevolved with humans through mutualistic interactions and performed fundamental roles in maintaining our physiological and metabolic homeostasis in response to changes in our intrinsic and extrinsic environments. However, other microorganisms assumed utterly invasive roles and became pathogenic to humans, thus posing health risks and threatening human survival. Ultimately, the outcome of competitive interactions between commensal members of our microbiota and potential pathogens would become a key life/death determining factor for human beings [49]. Recent methodological advances (e.g., high-throughput DNA sequencing) in paleomicrobiology [50,51,52] have allowed substantial progress in expanding our understanding on the appearance of human infectious microorganisms, their co-evolution with humans, the health conditions of past human populations, and the overall global ecological interactions across time. Accordingly, we know now that microbial pathogens, particularly bacteria, have been infecting humans for thousands of years [53,54,55]. It is currently estimated that the most recent common ancestors of *Helicobacter pylori*, which infects human stomachs, dates to the time of appearance of the anatomically modern humans [53]; *Mycobacterium tuberculosis*, the etiological agent of tuberculosis, has been around for less than 6000 years [53,54] and *Yersinia pestis*, which caused the plague, has spread globally for at least 5000 years [53,55]. Data derived from paleomicrobiology along with written historical records have clearly shown that, at times, certain pathogenic bacteria spread at high rates and their transmission acquired epidemic or even pandemic proportions (Table 1). However, it has become also clear that the progression of human civilization to more sedentary ways of life (e.g., the transition from hunter-gatherer communities to societies with agriculturalist and pastoralist economies) followed by the creation of ever larger cities and the establishment of better ways of communication between cities allowed the appearance of sustained infections by human-adapted bacterial pathogens [56,57], many of which were of zoonotic origin, transmitted from animals in various ways [58,59,60].

Information about the treatment of bacterial infections through the early years of the 20th century has been recorded in writings from ancient Greece, where Hippocrates became the founder of modern medicine [61,62]. With his conception of disease as an imbalance affecting the four basic bodily fluids (or “humors”) and of treatments as ways to help nature restore the lost balance, Hippocrates influenced the way clinical practice was carried out through the centuries [63] essentially globally and all the way to the modern world. Control and treatment of diseases involved the use of procedures such as bloodletting, dietary interventions, consumption of laxatives, management of rest and exercise, and others. Bacterial infections were no exception and, with the inclusion of amputations in extreme cases, were treated according to the principles of the “humor theory” until the discovery of penicillin by Alexander Fleming in 1928 and its clinical availability in the late 1930s [64,65]. The beneficial effects of penicillin were immediately appreciated, as its use saved thousands of lives during WWII and resulted in a substantial increase of the human life expectancy in a rather short time. However, even as early as 1945 there were already warnings about the need for a sensible use of penicillin by both patients (to avoid self-medication) and by physicians (to use appropriate dosing protocols) as there were already some signs of the development of penicillin resistance. Unfortunately, the reaction of many physicians and scientists to solve the resistance problem was to focus on the identification and isolation of novel antibiotics, rather than concentrating on introducing corrective behavioral measures. Although, initially, the use of additional newer antibiotics cured many bacterial infections, antibiotic resistances continued to rise and, at some point, no antibiotics were available to treat certain infections (e.g., MRSA, methicillin-resistant *Staphylococcus aureus*; MDR-TB, multidrug-resistant *Mycobacterium tuberculosis*) [66]. After many years with no new antibiotics available, efforts are currently on the way to discover compounds that may exert their antibiotic activity through pathways less favorable for the development of resistance mechanisms by the targeted bacterial populations [67,68]. The introduction of other kinds of compounds (e.g., bacteriophages, enzybiotics, and others) as alternatives to the use of antibiotics that do not lead to drug-resistance effects will be discussed later in this review.

## 2. Bacteria and Cancer

There are a number of converging associations between prokaryotic microorganisms, particularly bacteria, and the incidence of cancer in the animal kingdom, including humans [69,70]. Similar to the case of infectious diseases, written records from different cultures [71,72,73,74] and paleo-oncology/pathology data [75,76,77,78] clearly show that cancer is indeed an ancient disease (Table 2). In fact, the earliest hominid cancer described to date corresponds to a 1.7 million-year-old osteosarcoma case characterized in South Africa [79]. Interestingly, similarly to bacterial infections, cancer treatments were also based on the “humor theory” through the mid-19th century, with progressive incorporation of improved surgical techniques [80,81]. It was not until the 1890s that X-rays were used as the first form of radiotherapy [82], around 1940 for the beginning of chemotherapy [83], after 1970 for the use of antibodies [84], and much more recently for the use of protocols targeting the immune checkpoints [85], bringing immunotherapy to the first line of currently available anticancer therapies. It is worth mentioning this point that the term “chemotherapy” was first introduced in the early 1900s by the German biochemist Paul Ehrlich to refer to the use of chemicals to treat diseases, in particular infectious diseases [83]. Currently, the term chemotherapy is most frequently understood as referring to the use of chemicals for the treatment of cancer. Nevertheless, the concept seems to be appropriately interchangeable between anticancer and antimicrobial treatments, because diverse antibiotics with activity as DNA alkylating agents (e.g., Adriamycin, also called doxorubicin, and other anthracyclines produced by *Streptomyces* spp.) are used in anticancer regimens [83], and various drugs used in cancer chemotherapy (e.g., cisplatin, which is still used today as the standard of care for human cancers such as testicular tumors) are also known to have antimicrobial activity [86].

Most likely, the most direct association between bacteria and human cancer derives from the fact that certain bacterial infections cause cancer [87]. Bacteria induce carcinogenesis through two main mechanisms: (a) the induction of chronic inflammatory processes leading to cancer in various human organs, or (b) the production of carcinogenic metabolites, which is frequently the case for colon cancer [87]. Perhaps the best known instance of bacteria-induced cancer is that of *Helicobacter pylori*, which causes gastric MALT (mucosa-associated lymphoid tissue) lymphoma. As the *H. pylori* infection can spread by contaminated food or water and is transmitted by mouth-to-mouth contact, it is frequently acquired during childhood, and it is estimated to be present in over 60% of the world’s population, being particularly frequent in developing countries [87]. Prolonged *H. pylori* infection ultimately leads to chronic inflammation, a process that dramatically changes the gastric mucosa and stimulates regenerative cell proliferation as well as the production of reactive oxygen species (ROS) and of reactive nitrogen species (RNOS), which together may result in point mutations, deletions and/or translocations in the DNA of the host cells, thereby triggering the onset of the carcinogenic process [88]. The possible association of *H. pylori* with other types of cancer such as those in the colon [89], lung [90,91] and pancreas [90,92] has also been reported. Fortunately, treatment with antibiotics alone or in combination with agents that may prevent the development of antibiotic resistance can eradicate the *H. pylori* infection and, consequently, prevent the development of cancer [93,94,95].

A third point of convergence between bacteria and cancer relates to the role, mentioned above, of the human microbiota as a global homeostasis regulator by which it provides protection against a number of diseases, including cancer. After carcinogenic exposures, a well-balanced microbiota, with regard to both the strain diversity and the relative size of the various bacterial components, could be a key determinant of the outcome of the pro-carcinogenic process, allowing the onset of tumor formation or not. In this scenario, although a small percentage of cancer cases have a genetic component [96], it is important to consider the fact that cancer is an environmental disease [97,98], and cancer risk can be possibly influenced by extrinsic factors (e.g., diet, pollutants, carcinogens, or lifestyle) as well as by intrinsic factors (e.g., epigenetic signaling, microbiota composition, detoxifying proficiency, or immune competence). In this context, antibiotics are the kind of compounds that have the potential of modifying cancer risk due to their ability to act as extrinsic environmental chemical carcinogenic factors (direct action) and to alter the normal balance of the microbiota towards a more pro-carcinogenic composition (indirect action). The obviously beneficial action of antibiotics against bacterial pathogens may be, therefore, outweighed by the possibility of inducing a parallel increase in cancer risk.

**Figure 1:**
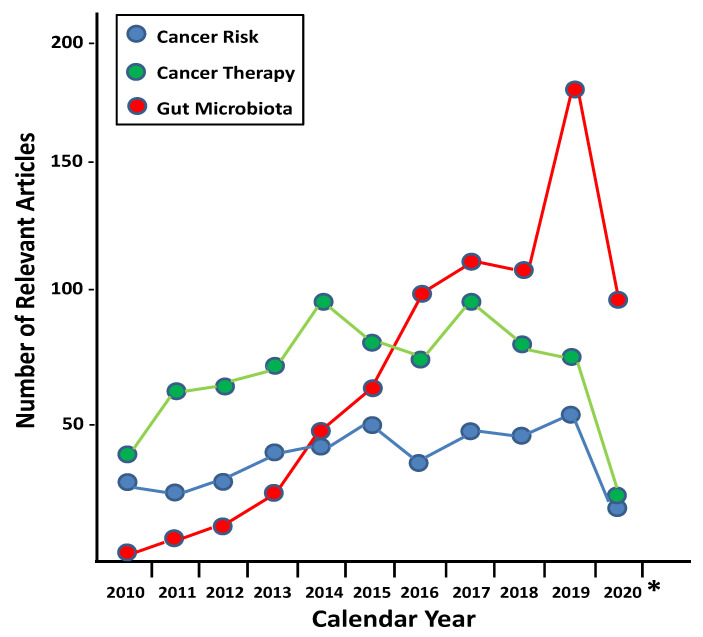
Record of publications over the last 10 years on the direct effects of antibiotic exposure on cancer risk (blue) and therapy outcomes (green), compared to microbiota-mediated studies (in red). Information for 2020 only includes data for the first six months of the year (*).

The significance of this issue is highlighted by (a) the over-exposure of humans to antibiotics through self-medication or by sharing prescriptions with friends or relatives in many communities, regions and countries where antibiotics can be accessed without prescription [99], a societal problem that can be easily solved with appropriate guidance [100]; and (b) by misguided indication and inappropriate dosing schemes prescribed by physicians and medical institutions [101,102] for the treatment of clinical cases not even involving bacterial infections, in many cases to simply satisfy the demands of patients to be prescribed “anything” to sense that they are adequately taken care of. In addition to these instances of uninformed, unnecessary, unjustified and inadequate antibiotic use that still make us question the safety of antibiotics in the 21st century [103,104], cancer patients are being exposed frequently to antibiotics as prophylactic or therapeutic components of their anticancer treatment protocols, particularly during postoperative periods after surgery [105,106,107] as well as while patients are immunosuppressed by the action of chemotherapeutic drugs [108,109]. In addition, it is possible that, either through their own direct carcinogenic action or by indirectly modifying the microbiota, antibiotic exposure may alter the response of cancer patients to therapy by lowering its effectiveness, thereby resulting in the appearance of secondary cancers, the progression to advanced stages, including metastasis or tumor recurrence. These two aspects of the possible influence of antibiotics on cancer risk and therapeutic outcomes have been studied from two main points of view: epidemiological analyses directly studying the association between antibiotic exposure and cancer incidence, and evaluations of the possible involvement of indirect antibiotic effects on the microbiota in promoting cancer onset and development. As evidenced by publication records over the last 10 years (Figure 1), results from the latter type of studies have been, and continue to be, reported at a higher frequency than the epidemiological data. The next sections will examine the connection between antibiotic exposure and cancer risk and its effects on treatment effectiveness and outcomes for cancer patients.

## 3. Antibiotics and Cancer Risk

As correctly expressed by McCormack and Boffetta in the title of one of their articles (“Today’s lifestyles, tomorrow’s cancers: trends in lifestyle risk factors for cancer in low- and middle-income countries”) [110], the reality is that it is precisely in those countries where not only the unregulated consumption of antibiotics happens more frequently, but also where, unlike what happens in developed counties [111], accurate records of cancer incidence are not periodically updated or not even maintained at all. Given this situation, epidemiological assessments about antibiotic exposure and cancer risk are very valuable. In the course of the last fifteen years, studies on possible effects of antibiotic exposure on cancer risk have focused primarily on the cancer types more frequent in humans, and generally have been designed to include cohorts of cancer patients and randomly selected non-cancer patients as controls.

In studies related to breast cancer, the data suggested an association between antibiotic consumption and cancer risk. Although in some studies the association was qualified as weak [112,113], other studies reported a clearly positive association with the number of prescriptions and the cumulative days of antibiotic use [114,115]. While in some studies the same patterns of association were observed with all classes of antibiotics tested [114,115], a better association was reported by different antibiotic classes [112,116]. The situation was not clear with regard to lung cancer, as the data provided insufficient evidence to support or refute a possible carcinogenic effect of antibiotics [117]. The information from studies on colorectal cancer (CRC) seems more conclusive, most likely due to the greater number of studies published much more frequently because of the general trend of increased scientific interest in the gut microbiota. Most CRC-related studies report an association, even at the adenoma stage, with both timing and duration of antibiotic exposure [118,119,120]. In addition, and more importantly, some of these studies allowed the dissociation between the effects of antibiotic usage on the risk of colon cancer vs. rectal cancer, as the data consistently showed a positive association between antibiotic use and colon cancer, but there was either no association or a negative correlation with cancer of the rectum [121,122,123].

In more general studies of other digestive cancers (esophagus, stomach, small intestine, hepatobiliary, and pancreas), positive associations were found between certain antibiotic classes and particular tumor types, which increased with dose [124,125]. Positive associations were found between the use of penicillins and esophageal, gastric and pancreatic cancer, with clearer dose-response effects in the latter type [124]. Nitroimidazoles and quinolones showed more modest associations with all digestive tumor types investigated [125]. Studies on non-melanoma skin cancer showed that there was an increased risk of developing skin cancer associated with the use of photosensitive antibiotics [126,127,128,129,130]. Exposure to antibiotics such as ciprofloxacin, ketoconazole, and sulfamethoxazole increased the risk of developing basal cell carcinoma (BCC), whereas the use of doxycycline and sulfamethoxazole increased the risk of squamous cell carcinoma (SCC) [126,127,129]. Although some studies associated the use of tetracycline with BCC risk [126,127], it was also reported that the use of tetracycline demonstrated positive interactions regarding simultaneous UV light exposure and the risk of SCC [129]. An association was also observed between the use of moxifloxacin and an increased risk of developing SCC during the first year after lung post-transplantation [128]. In addition, the use of a mathematical model also predicted, and somehow confirmed, that the risk of developing skin cancer is positively associated with the use of antibiotics [130]. Finally, two large multi-tumor type studies [131,132] are worthwhile mentioning. In the first one [131], researchers followed for a period of six years the number of cancers diagnosed in a sample of 3,112,624 individuals with no previous history of cancer, and analyzed that information with regard to the patterns of antibiotic usage in the study population. Data from this study showed that cancer incidence increased with the number of prescriptions, and that the extent of the association of the relative risk with antibiotic exposure varied with tumor type, being greatest in tumors of endocrine glands, followed in decreasing order by cancers of the prostate, breast, lung, colon and ovary [131]. The second multi-tumor type study [132], the largest reported to date, reported results from the systemic review of about 7.9 million individuals showing that, on average, antibiotic use increased cancer risk by about 18%, although the effect varied with tumor type: 30% increased incidence of lung, pancreatic and genitourinary cancers; smaller risk increases (6–8%) for CRC, gastric cancer and melanoma; and no association was found with esophageal or cervical cancer. With regard to antibiotic types, the highest risk was associated with the use of β-lactams, cephalosporins and fluoroquinolones [132].

## 4. Antibiotics and Cancer Therapy Outcomes

A number of reports have been published during the last few years on the possibility that the prophylactic antibiotic treatment of cancer patients, which is deemed a necessary approach to prevent infections after surgery or during chemotherapy, may affect the outcome of their cancer treatment. After early encouraging reports showing that the use of anthracyclines such as doxorubicin, epirubicin or idarubicin to treat various tumor types resulted in the potentiation of the patient’s anti-tumor immunity [133], and data from other studies showed that antibiotic treatment had no deleterious effects on the response of non-small cell lung carcinomas (NSCLC) to treatment with the immune checkpoint inhibitor (ICI) nivolumab [134,135], results from preclinical chemo-immunotherapy protocols combining cyclophosphamide chemotherapy with adoptive T-cell (ACT) immunotherapy, using a mouse model of B-cell lymphoma, demonstrated that prophylactic use of broad-spectrum antibiotics reduced the efficacy of cyclophosphamide and impaired the therapeutic effects of ACT [136]. Since then, most studies have reported negative effects of antibiotic exposure leading to diminished levels of efficacy of ICIs in immunotherapy protocols for the treatment of a variety of tumors, including lung tumors/NSCLC [137,138,139,140,141,142,143,144], advanced or metastatic renal cell carcinoma [137,141,142,144], urothelial carcinoma [141], and melanoma [141,143,144]. In addition, more recently, it has been reported that antibiotic use had a negative impact on the response of patients with locally advanced head-and-neck tumors to treatment protocols involving chemotherapy or radiotherapy [145]. The abundance of reports describing the negative effects of antibiotic exposure on the response of cancer patients to different types of therapeutic modality strongly suggests that the final outcome may be related to a unifying element, and that such element is the state of intrinsic microbiota.

## 5. Central Regulatory Role of the Microbiota

As indicated above, over the last ten years there has been an extraordinary interest in the human microbiota, in particular with regard to its critical ability to maintain the health/disease balance through our lives. It is now clear that the microbiota plays a number of central regulatory roles related to environmental risks [146], antibiotic response [147], tumor progression [148], or the response to cancer therapy [149,150,151]. The basic mechanisms by which microbiota imbalances stimulate cancer development can be divided into two broad types: genetic and epigenetic. Genetic mechanisms relate to inducing DNA damage, interfering with the DNA-damage response and, consequently, leading to the accumulation and transmission of mutations in the host DNA. In addition, microbiota dysbiosis has substantial epigenetic effects, including changes in global DNA methylation, histone acetylation, chromatin remodeling and other epigenetic abnormalities [152]. Microbiota imbalances trigger some of these genetic and epigenetic pro-carcinogenic effects through enzymes, toxins, metabolites such as short-chain fatty acids (SCFAs), or other products either secreted by gut microbes or generated as byproducts from their metabolic conversion of dietary components and other ingested xenobiotics [152]. It is through interactions with cellular receptors and cell signaling cascades that the microbial-derived secreted or metabolic products mediate the microbiota’s positive or negative, in modulating the therapeutic response of cancer patients. As a target for antibiotics action [153], it seems highly likely that diverse levels of microbiota dysbiosis may boost the negative effects of antibiotic exposure on both the enhancement of cancer risk and the efficiency dampening of chemotherapy, immunotherapy and radiotherapy protocols. In this context, it seems quite obvious that the introduction in the clinic of strategies that may allow microbiota modulation may be a key step towards providing an optimum biological framework to facilitate cancer prevention and treatment [154]. The decrease in gastric cancer attained by eradicating *H. pylori* is a clear example of the anticancer potential of microbiota modulation [155]. It seems, therefore, possible that taking into consideration the status of their microbiome may improve the use of antimicrobial agents, as well as the treatment outcomes for cancer patients [156]. Protocols for microbiota modulation may provide tools to achieve predictive information on cancer treatment outcomes [157,158,159].

## 6. Alternative Approaches

On the basis of the information presented above, the most urgent issue is to promote a change in the patterns of antibody utilization [160] as a way to decrease not only cancer risk, but also the increased risk for all causes of mortality associated with long-term antibiotic use in late adulthood [161] as well as possible risks passed to the offspring during different stages of pregnancy [162]. Alternatively, efforts should be made to identify or develop new antibiotics that may have both antimicrobial and anti-tumor activities [163,164]. The selection of antibiotics with anti-tumor activity may be done by taking into consideration: (a) their mechanism of action, by taking advantage of properties such as their genotoxicity [165,166], their apoptosis-inducing potential [167,168], their ability to block tumor-specific signaling pathways [169], their epigenetic modulatory effects [170,171], or other relevant molecular mechanisms [172,173]; and (b) the lowest possible deleterious effects on the microbiota, as in the case of rifaximin [174,175], that has broad-spectrum against both Gram-positive and Gram-negative bacteria and, on the basis of its unique absorbability, solubility and pharmacokinetic properties may in fact correct microbiota dysbiotic imbalances [176].

Microbiota modification techniques provided an obvious second main alternative, which can be accomplished by the use of probiotics, prebiotics or symbiotic supplements [177,178] or, more directly, by fecal microbiota transplantation (FMT) [179,180]. Currently available FMT methods [181] make it possible to reverse dysbiotic processes of the microbiota [182] as well as provide, at the same time, new opportunities for improved cancer management protocols [183].

Finally, there is also the possibility of substituting antibiotics for other agents that may have similar advantages with regard to their antimicrobial activity, but do not create collateral problems related to resistance, microbiota dysbiosis, or the decrease in the response to anti-tumor therapies. Although agents with dual antimicrobial and anti-tumor activities would be ideal, combined applications of mono-therapeutic agents may easily provide the same effect. Two types of agents to be considered in this class are bacteriophages [184,185,186,187] and enzybiotics [188,189,190,191], which have attracted substantial attention in recent times, and can be used in prophylactic and therapeutic applications in antimicrobial and anticancer protocols. Bacteriophages have been engineered for medical applications [192] in ways allowing them to retain their antibacterial activity [185] and have been used as anticancer drug delivery systems [186,192], their possible effects on anti-tumor immunity and the response to anticancer therapy must be further evaluated. The same is even more needed with regard to enzybiotics, poorly immunogenic enzymes from bacteriophages or other natural origin able to highly specifically act as antimicrobial agents [187], as our current knowledge about their potential use in anticancer strategies is extremely limited.

## 7. Conclusions and New Perspectives

**Figure 2:**
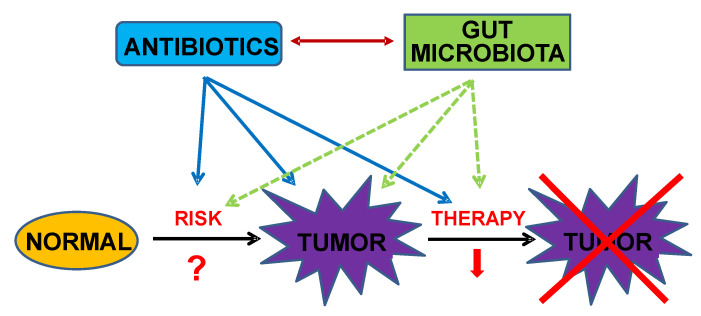
Diagram showing the interactions between antibiotic exposure and the human gut microbiota, and the possible effects of their cross-talk on the risk of cancer development and the response to therapy are indicated by arrows.

Interactions between antibiotics and the microbiota regulate their respective contributions to the carcinogenic process (by modulating cancer risk and tumor initiation) and to the response of cancer patients to different anticancer therapies, leading to effective cures or to progression of tumors to advanced, metastatic stages. Figure 2 illustrates this dual regulatory interaction. Alternatives to the use of antibiotics that either do not cause or only cause minor levels of microbiota dysbiosis provide potentially useful strategies to keep the carcinogenic process under control. However, there is another factor that must be taken into consideration: the human tumor microbiome.

The fact that bacteria found in human tumors were not the result of contamination was established about 100 years ago, and it has been only recently that technological advances have allowed the establishment of tumor microbiome signatures as distinct from those derived from genomic analyses of the normal microbiota. Nevertheless, detailed characterization of the tumor microbiota has not progressed at a fast pace, due to the limitations imposed by the very low bacterial biomass present in the tumors. Despite this situation, a very exciting finding has been very recently [193] reported demonstrating that different tumor types have distinct microbiome signatures, which has important implications from a diagnostic point of view, and even more importantly that the tumor microbiota is composed of intracellular bacteria. Understanding the contribution of tumor type-specific, intracellular bacteria to the balance of the normal microbiota and the effects of antibiotics in the context of cancer risk and therapy efficiency will definitely require the application of system biology approaches [194].

Interestingly, this finding brings us back to the world of prokaryotes that provide specific functions while being intracellular residents, as a possible case of what could be called “oncologic symbiosis”. Furthermore, the intracellular location of the tumor microbiota connects it to some of the most recent theories on the origin of tumors [195,196]. One of these notions sees the roots of cancer etiology as grounded in the two transitions (from prokaryotic to eukaryotic, and from unicellular to multicellular beings) that ultimately lead to the establishment of higher organisms [195]. The other, the so-called “Systemic-Evolutionary Theory of Cancer (SETOC)”, proposes that, as a consequence of long-term injuries caused by cancer-promoting factors, cancer results from a process of regression of the eukaryotic cells towards a situation in which its prokaryotic component assumes uncoordinated behaviors, which ultimately break the integration of the components of the endosymbiotic cellular system [196]. It will be extremely interesting to see whether the newly identified intracellular prokaryotic cells of the tumor microbiota play any role in these proposed regressive processes.

## Figures and Tables

**Table 1:** Records of widespread human bacterial infections.

Time Period	Main Bacterial Agents(s)	Geographic Distribution
5000–1500 BC	*Yersinia pestis* *Helicobacter pylori*	**Paleomicrobiology** records suggest frequent infectious diseases
XIV Century BC	*Francisella tularensis*	The **Hittite Plague** was most likely a case of tularemia, a zoonotic, possibly fatal disease in humans, that spread through the Middle East
430–426 BC	*Salmonella enterica*, serovar *Typhi* identified as a possible cause	**Plague of Athens**, ancient Greece, later spread through war with infected animals to a wider geographical region
541–544 AD	*Yersinia pestis*	**Justinian Plague**, spread through Asia, North Africa, Europe and the Arabian Peninsula
1347–1351 Later outbreaks: 1616–1619 1629–1631 1656–1658 1665–1666 1720–1722	*Yersinia pestis*	**Black Death**—Bubonic plague, widely spread through Europe and Asia **Massachusetts Plague**, North America **Italian Plague**—Milan —Naples **Great Plague of London** (England) **Great Plague of Marseille** (France)
1817–1824	*Vibrio cholerae*	**Cholera epidemic**—India, China and Southeastern Asia
1894	*Yersinia pestis*	**Bubonic plague**—India and China
1899–1923	*Vibrio cholerae*	**Cholera pandemic**—Started in India and spread over the years to the Middle East, North Africa, Eastern Europe and Russia
1994	*Yersinia pestis*	**Indian Bubonic Plague**

**Table 2:** Brief history of cancer.

Time Period	Civilization(s)	People/Events
2500–1500 BC ca. 1825 BC ca. 1538 BC	Ancient Egypt	Earliest descriptions **Medical Papyri** *Papyrus Cahun* *Papyrus Ebers*
1400–1100	Chinese	Oracles written to provide earliest documentation on cancer cases
475–221 BC	Chinese	**Writings** “*Inner Cannon of Yellow Emperor*” on etiological factors (e.g., diet, depression, body deficiencies), symptoms and pathology “*The Classic Mountains and Seas*” on treatments with different types of seaweeds.
460–310 BC	Greek	**Hippocrates** Described several cancer types, with drawings Coined the term “*karkinos*” (for “crab”) based on the appearance of tumors Treatments based on “Humor Theory” (diet, bloodletting, laxatives)
25 BC–50 AC	Roman	**Celsus** Coined the term “*cancer*” (Latin for “crab”) Cancer was appreciated as being common enough to be widely studied and recorded
130 AD–210 AC	Greece	**Galen** Coined the term “oncos” to refer to the swelling associated with all tumors Recognized the differences between malignant (“karkinos”) and non-malignant tumors Use the suffix “-oma” (still used for tumor types) Established modern concept of *Oncology*
III–VII Century AC	Western Europe	**Medical Handbooks** (*Orebasius*, *Aetios of Amida*, *Paul of Aegina*) compiled with more detailed descriptions and drawings of various tumor types
648 AC	Chinese	Surgery used for the first time to remove tumors
VII–XIV Century AC	ARAB and MuslimCultures	Scholars (**Avicenna, Rhazes, Al Zahrawi, Ibn al Nafis**), mainly in the Caliphate of Cordoba (what is now Spain) made important advances: Invention of surgical tools First removal of early-stage breast cancer Realization that successful treatment was possible if detected early
XV–XVIII Century AC	European	**Avicena’s** “*The Cannon of Medicine*” remained the Standard in cancer management
XVIII Century AC to Present ca. 1940 ca. 1970 recently	Worldwide	Advances in surgical techniques Discovery of Radiation and therapeutic use of X-rays First use of Chemotherapeutic protocols First use of Immunotherapy approach Introduction of Immune Checkpoint Inhibitors
